# A new journal and new global perspective on infection control and public health

**DOI:** 10.1186/2047-2994-1-4

**Published:** 2012-01-26

**Authors:** Andreas Voss, Jan Kluytmans, Didier Pittet

**Affiliations:** 1 Canisius-Wilhelmina Hospital, Regional Infection Control Centre (i-Prevent), PO Box 9015, 6500 GS, Nijmegen, The Netherlands; 2 Radboud University Nijmegen Medical Centre, Department of Medical Microbiology, 6500 HB, Nijmegen, the Netherlands; 3 Laboratory for Microbiology and Infection Control, Amphia Hospital Breda, PO Box 90158Breda, 4800 RK, The Netherlands; 4 Department for Microbiology and Infection Control, Free University Medical Centre, PO Box 7057, 1007 MB Amsterdam, The Netherlands; 5 Infection Control Programme and WHO Collaborating Centre on Patient Safety, University of Geneva Hospitals and Faculty of Medicine, 4 Rue Gabrielle Perret-Gentil, Geneva, Switzerland; 6 Global Patient Safety Challenge "Clean Care is Safer Care", WHO Patient Safety, WHO Headquarters, Avenue Appia, 1211 Geneva 27, Switzerland

## 

With the many changes in healthcare, the occurrence of new and emerging infectious diseases, and pandemic of novel resistance mechanisms, the prevention of healthcare-associated infections (HAI) has become increasingly important. Borders are disappearing - not only between different healthcare settings locally or nationally - but also between developed and resource-limited countries, thereby transforming the fight against HAI into a truly global challenge. This change is reflected by the World Health Organization's (WHO) choice of an infection control topic for the First Global Patient Safety Challenge - *Clean Care is Safer Care *- and the strong focus of the diagnostic and biomedical industry on HAI prevention and the control of antimicrobial resistance (AMR).

According to WHO, hundreds of millions of patients develop HAI every year worldwide and as many as 1.4 million occur each day in hospitals alone [1]. The burden of disease is obviously much higher in low- and middle-income countries [2], but it is a challenge in itself to provide reliable global estimates and data are scarce from regions of the world where resources are the most limited [3]. Nevertheless, it is clear that a great number of global citizens suffer from preventable HAI. HAI are associated also with huge costs that are no longer affordable for most healthcare systems, including those in resource-rich countries.

AMR and the emergence of new pathogens and/or re-emergence of old ones are influenced by a large variety of factors with many far beyond the boundaries of human medicine and local or even national practices. Pathogens that were initially strictly hospital-acquired, such as methicillin-resistant *Staphylococcus aureus *(MRSA), became community pathogens (CA-MRSA) and even zoonotic pathogens with the emergence of livestock-associated (LA)-MRSA, thereby totally changing the epidemiology of MRSA. CA- and LA-MRSA even pose a serious challenge to the long admired "search and destroy" strategy. Many see the local or national prevalence of MRSA as a surrogate marker for the quality of infection control, and the increasing efforts of nations to address the challenge of ever-increasing MRSA rates seem to produce the right effect [4,5]. While certainly continuing to be a major challenge, the tide seems to be changing from Gram-positive to Gram-negative threats.

Multidrug-resistant Gram-negatives expressing extended-spectrum beta-lactamases (ESBLs) or carbapenemases are already highly prevalent both in the community and the environment. Recent studies described that most pieces of retail meat contained ESBL [6]. In addition, New Delhi metallo-beta-lactamase-1 (NDM-1) was isolated from drinking water [7]. This creates an unprecedented, large transmission route and, consequently, the need to develop new infection control strategies. The resistance mechanisms involved are not bound to the DNA of a bacterial species, but are a transferable genetic element (TGE). Not a superbug, but a super-TGE![8] The emergence of TGEs not only increases the opportunity for spread, but requires new techniques to trace the spread of resistance. Traditional typing methods are limited to the spread of a strain and cannot be used to show the spread of TGEs. The potential for the spread of multidrug-resistant Gram-negatives seems unlimited. For example, more than 80% of travellers to India or Pakistan return with ESBLs in their gastrointestinal tract. These individuals may become a source of transmission in their home country due to the potential extended carriage of ESBLs. Indeed, 20% will be still positive 6 months after their return [9]. The global population today is highly mobile and problems arising in faraway countries can no longer be ignored, if we intend to control the spread of these pathogens.

There is now a pressing need to share knowledge and experience in HAI prevention and control of AMR with colleagues from countries and cultures around the world. This is well reflected by the success of the recent 1st International Conference on Prevention and Infection Control (ICPIC 2011) held in Geneva, Switzerland, under the auspices of the WHO Collaborating Centre on Patient Safety. ICPIC was initiated to create a truly international platform for the exchange of knowledge, best practices, and cutting-edge research from around the globe (http://www.icpic.com). With more than 1200 participants from 84 nations, the first edition of ICPIC provided solid proof that many of our colleagues around the world share our view. An impressive delegation from several African countries contributed to creating solidarity between developed and developing countries. In the past, research and continuing education were driven by a few North American and European centres, while today a growing number of centres from around the globe join ever-increasing efforts to develop measures for the prevention of HAI and AMR spread. Many were represented at ICPIC, which can be seen as a turning point. The world will need an increasing number of these centres to provide expert knowledge and guidance on how to control HAI and resistance spread in diverse settings, health systems, and populations.

A logical consequence to ICPIC was to continue our vision by creating a new journal based on the same ideas. We are proud to announce the birth of *Antimicrobial Resistance & Infection Control *(*ARIC*), a truly international, online, open access journal. *ARIC *aims to be a global forum for all researchers working in the fields related to the prevention, diagnosis, and treatment of HAI and AMR development in all health-care settings, systems, and populations worldwide. A better understanding of factors contributing to the development and spread of multidrug-resistant pathogens, possibilities to prevent transmission and infections, and insight into the difference between developed countries and countries with limited resources are key factors to delineate future solutions. *ARIC *intends to cover a broad spectrum ranging from preeminent practices and best available data to the best interventional and translational research and innovative (technical) developments in the field of infection control and AMR to overcome the challenge posed by HAI.

We are convinced that *ARIC *will become the future global forum for reports encompassing all aspects of resistance development and prevention of HAI across all health-care settings. Prevention of HAI in hospitals, infection control and AMR in high-risk settings (e.g. ICUs), HAI prevention and AMR in specific settings e.g. long-term care facilities or community settings, special problems with infection control and AMR in resource-limited countries, and knowledge transfer from developed to developing and from developing to developed countries [Syed SB, Dadwal V, Rutter P, Storr J, Hightower JD, Gooden R, Carlet J, Bagheri Nejad S, Kelley ET, Donaldson L, Pittet D. **Developed-developing country partnerships: benefits to developed countries**. Lancet 2011 (submitted).] are just a few examples of what we hope to cover.

ARIC's choice of "open-access" is a logical step towards its goal to be truly international and to allow the transfer of knowledge and best practices to even remote places that cannot afford printed journals. We realize that open access has financial consequence for some authors and that the standard BioMed Central rules to waive article-processing charges may not be enough. BioMed Central provides an automatic waiver to authors based in any country classified by the World Bank as low-income or lower-middle-income economies[10], but we intend to find solutions in the near future that will allow us to support authors from upper-middle income countries and young investigators.

We look forward to welcoming many papers and to developing this journal with your support into *the *online forum for sharing best practices, knowledge, and latest research with colleagues from around the world.

Andreas Voss - Editor-in-Chief, *ARIC*

Jan Kluytmans- Deputy Editor, *ARIC*

Didier Pittet - Executive Editor, *ARIC*
